# Non-ST Segment Elevation Myocardial Infarction (NSTEMI) in the Setting of Severe Rhabdomyolysis and COVID-19 Infection: A Case Report

**DOI:** 10.7759/cureus.40554

**Published:** 2023-06-17

**Authors:** Mubariz A Hassan, Yashvardhan Batta, Tori Smith, Muhammad Adil Afzal

**Affiliations:** 1 Internal Medicine, Howard University Hospital, Washington, USA; 2 Internal Medicine, St. Joseph's Regional Medical Center, Paterson, USA

**Keywords:** cardiac troponin, acute kidney failure, covid-19, covid-19 with rhabdomyolysis, non-st segment elevation myocardial infarction (nstemi)

## Abstract

We present a case report of a non-ST segment elevation myocardial infarction (NSTEMI) occurring in an 89-year-old male with severe rhabdomyolysis and COVID-19 infection. The patient had a complex medical history, including non-ischemic cardiomyopathy, sinus bradycardia status post permanent pacemaker placement, and multiple comorbidities. He presented to the emergency department after a mechanical fall and was found to be COVID-19 positive. Despite the absence of typical symptoms, the patient's elevated troponin levels and electrocardiogram findings indicated NSTEMI. The initial management included an acute coronary syndrome protocol and admission to the cardiac intensive care unit. During the hospitalization, the patient developed acute hypoxic respiratory failure and was treated for COVID-19 pneumonia. The patient's renal function and creatine kinase levels showed improvement, and cardiac catheterization revealed non-obstructive coronaries. The patient was discharged in stable condition with a follow-up scheduled.

## Introduction

Non-ST segment elevation myocardial infarction (NSTEMI) is a clinical entity characterized by myocardial ischemia and infarction without the presence of ST-segment elevation on electrocardiography. It can occur in various clinical settings, and its management requires a comprehensive approach. In the context of severe rhabdomyolysis and COVID-19 infection, the diagnosis and management of NSTEMI present additional challenges. Rhabdomyolysis can lead to myocardial injury due to the release of myoglobin and other cellular contents, while COVID-19 infection has been associated with increased cardiovascular complications [1]. Understanding the interplay between these conditions and their impact on the management of NSTEMI is crucial for providing optimal care to patients.

In this case report, we describe the clinical presentation, diagnostic evaluation, and management of an 89-year-old male with NSTEMI in the setting of severe rhabdomyolysis and COVID-19 infection. We highlight the importance of recognizing atypical symptoms and considering myocardial infarction in high-risk patients, even in the absence of typical chest pain or shortness of breath. The management approach involved a combination of acute coronary syndrome protocols, COVID-19-specific treatments, and supportive care for rhabdomyolysis and acute kidney injury (AKI). Additionally, we discuss the challenges in diagnosing and managing NSTEMI in the presence of concurrent COVID-19 infection and the implications for patient outcomes.

This case underscores the need for a multidisciplinary approach to address the complex interactions between different clinical conditions in patients with NSTEMI. By presenting this case, we aim to contribute to the existing literature and enhance the understanding of the interplay between severe rhabdomyolysis, COVID-19 infection, and NSTEMI, ultimately guiding the management strategies for similar cases in the future.

## Case presentation

The patient is an 89-year-old male with a complex medical history including non-ischemic cardiomyopathy, sinus bradycardia status post permanent pacemaker placement, benign prostatic hyperplasia with obstruction, normocytic anemia, stage IIIB chronic kidney disease, nephrolithiasis, gout, multiple strokes, hypertension, and obesity. He presented to the emergency department following a mechanical fall and a prolonged period of immobility. Notably, he tested positive for COVID-19 prior to presentation. Furthermore, there was no history of chest pain, shortness of breath, dizziness, or loss of consciousness. The patient also denies using alcohol or any other illicit drug use. Medication history was also negative for any statins or herbal medications.

**Figure 1:**
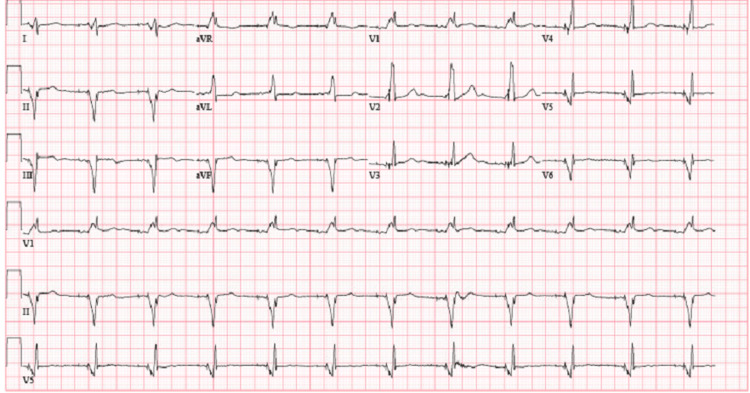
ECG showing atrial ventricular dual-paced rhythm

On exam, the patient was not in any acute distress with a temperature of 98.4°F, heart rate of 96 beats per minute, respiratory rate of 18 breaths per minute, blood pressure of 110/67 mmHg, and saturating 96% on 2 liters of oxygen via nasal cannula. The physical exam was unremarkable. Diagnostic investigations revealed several significant findings, including an electrocardiogram showing an atrioventricular dual-paced rhythm with no ischemic changes as shown in Figure 1. Elevated troponin and creatine kinase levels indicated myocardial injury, while brain natriuretic peptide levels were indicative of cardiac stress as mentioned in Table 1. The echocardiogram was performed, and it showed no regional wall motion abnormalities. Imaging of the head was negative for any acute abnormality and electroencephalogram (EEG) also ruled out any seizure disorder. Treatment was initiated following an acute coronary syndrome protocol, and the patient was admitted to the cardiac intensive care unit for the management of NSTEMI in the context of rhabdomyolysis, COVID-19, anemia, and AKI.

**Table 1:** Laboratory results

Basic Labs	Results	Reference Range
White Blood Cells	6.85	3.2-10.6x10^9^/L
Hemoglobin	8.3	14.6-17.8 g/dL
Hematocrit	25.7	40.8-51.9 %
Platelets	236	177-406x10^9^/L
Sodium	137	135-145 mEq/L
Chloride	105	95-111 mEq/L
Blood Urea Nitrogen	35	7-25 mg/dL
Creatinine	1.68	0.6-1.2 mg/dL
Potassium	4.3	3.5-5.1 mEq//L
Magnesium	1.92	1.7-2.5 mg/dL
BNP	507	<100 Pg/mL
Troponin (trend on admission)	3.50>5.59>5.95	<0.03 Ng/mL
Creatinine Phosphokinase CPK (upon admission)	15,980	35-230 IU/L
Creatinine Phosphokinase CPK (upon discharge)	401	35-230 IU/L

**Figure 2:**
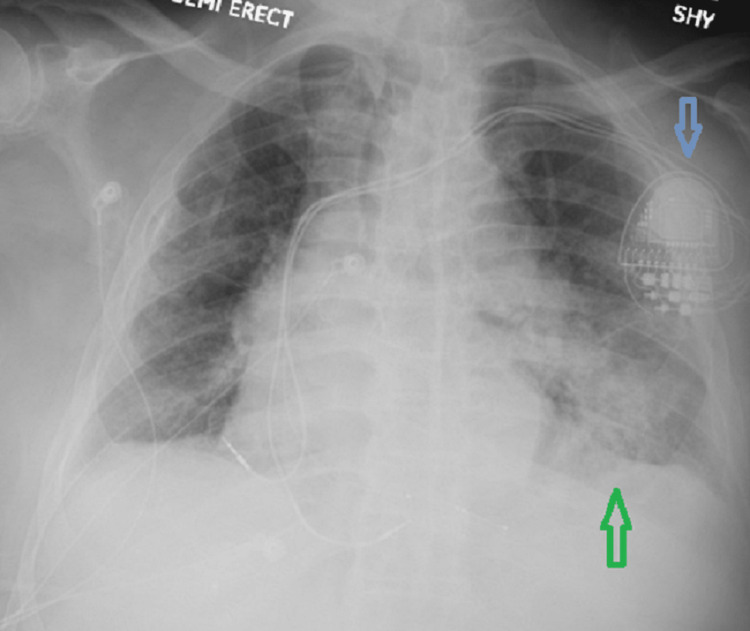
Anterior posterior view of portable chest x-ray (CXR) with blue arrow showing pacemaker and green arrow pointing toward the left lower lobe infiltrates

During second day of the hospitalization, the patient developed acute hypoxic respiratory failure, requiring supplemental oxygen with nasal canula and imaging revealed a left lower lobe infiltrate suggestive of COVID-19 pneumonia as shown in Figure 2. Appropriate interventions were implemented, including the administration of antiviral and antibiotic therapy, along with supportive measures. Additional investigations were conducted to rule out pulmonary embolism and deep vein thrombosis. The patient's respiratory status gradually improved, renal function stabilized, and cardiac catheterization demonstrated patent and non-obstructive coronary arteries. Subsequently, the patient was discharged in a stable condition with scheduled cardiology outpatient follow-up.

## Discussion

NSTEMI in the setting of severe rhabdomyolysis presents a unique clinical challenge due to the interplay between muscle injury and myocardial ischemia. Rhabdomyolysis, characterized by skeletal muscle breakdown, can lead to the release of intracellular contents, including myoglobin and other enzymes, into the systemic circulation.

NSTEMI is a type of acute coronary syndrome characterized by ischemic myocardial injury without ST-segment elevation on the electrocardiogram. NSTEMI can be further classified into different types based on the underlying mechanisms and etiologies. Type 1 NSTEMI is caused by the rupture or erosion of an atherosclerotic plaque leading to the formation of a thrombus, resulting in partial occlusion of the coronary artery. This type is typically associated with significant atherosclerotic disease and often requires invasive management strategies such as coronary angiography and revascularization [2]. Type 2 NSTEMI, on the other hand, occurs due to an imbalance between myocardial oxygen supply and demand in the absence of coronary plaque rupture. It is typically secondary to other conditions such as severe systemic illness, hypotension, tachycardia, anemia, or coronary artery vasospasm. In these cases, the underlying cause leads to myocardial ischemia without an acute thrombotic event. The management of type 2 NSTEMI involves addressing the underlying condition and optimizing the balance between myocardial oxygen supply and demand. The identification and management of the underlying etiology, such as sepsis, respiratory failure, rhabdomyolysis, or anemia, are crucial in type 2 NSTEMI cases [3].

The systemic effects of rhabdomyolysis can contribute to the development of NSTEMI through several mechanisms. The release of myoglobin can cause direct tubular injury in the kidneys, leading to AKI. AKI, in turn, can lead to volume overload, electrolyte imbalances, and impaired cardiac function. These factors can increase myocardial oxygen demand and precipitate myocardial ischemia in vulnerable individuals with pre-existing coronary artery disease or impaired coronary flow reserve [4].

The release of pro-inflammatory cytokines and oxidative stress associated with rhabdomyolysis can promote endothelial dysfunction and trigger a pro-thrombotic state. The activation of platelets and the coagulation cascade can lead to the formation of intracoronary thrombi or the progression of pre-existing coronary plaques, ultimately resulting in NSTEMI [2]. Furthermore, the systemic inflammatory response induced by rhabdomyolysis can contribute to endothelial dysfunction and microvascular dysfunction, impairing myocardial perfusion even in the absence of significant obstructive coronary artery disease. The compromised microcirculation can exacerbate myocardial ischemia and contribute to the development of NSTEMI [5].

The case presented here adds an additional layer of complexity with the concurrent presence of COVID-19 infection. Emerging evidence suggests that COVID-19 infection may contribute to an increased risk of cardiovascular complications, including myocardial injury and myocardial infarction [6]. The mechanisms underlying the association between COVID-19 and myocardial injury are multifactorial.

The direct viral invasion of cardiomyocytes through angiotensin-converting enzyme 2 (ACE2) receptors can lead to myocardial inflammation, injury, and dysfunction. This direct viral cardiotoxicity can contribute to the development of NSTEMI in individuals with COVID-19 infection, particularly in the presence of underlying risk factors such as rhabdomyolysis [7]. The systemic inflammatory response induced by COVID-19 infection, often referred to as the "cytokine storm," can exacerbate endothelial dysfunction, promote a pro-thrombotic state, and contribute to microvascular dysfunction. These effects can further compromise myocardial perfusion and increase the risk of NSTEMI [8].

Additionally, COVID-19 infection can contribute to a state of hypercoagulability and promote the formation of intracoronary thrombi, potentially leading to acute coronary syndromes such as NSTEMI [8]. The management of NSTEMI in the setting of severe rhabdomyolysis and COVID-19 infection requires a multidisciplinary approach. Prompt recognition of myocardial ischemia, optimization of cardiac function and hemodynamics, treatment of rhabdomyolysis, and appropriate management of COVID-19-related complications is essential. Furthermore, this case highlights the need for clinicians to remain vigilant and consider the possibility of cardiac involvement in patients with severe rhabdomyolysis and concurrent COVID-19 infection, even in the absence of typical symptoms or electrocardiographic changes. Timely evaluation and appropriate management are crucial to prevent further cardiac damage and improve patient outcomes.

## Conclusions

In conclusion, NSTEMI in the context of severe rhabdomyolysis and COVID-19 infection represents a complex clinical scenario with multiple underlying mechanisms. The release of myoglobin, pro-inflammatory cytokines, and oxidative stress associated with rhabdomyolysis, as well as the direct viral cardiotoxicity and systemic inflammatory response induced by COVID-19 infection, can contribute to myocardial injury and ischemia. This case report underscores the importance of a comprehensive and interdisciplinary approach in managing complex patients with overlapping medical conditions. Further research is warranted to better understand the underlying mechanisms and risk factors associated with NSTEMI in the setting of severe rhabdomyolysis and COVID-19 infection. This will help guide clinical decision-making and optimize treatment strategies for similar cases in the future.
